# Historical Aspects of Propolis Research in Modern Times

**DOI:** 10.1155/2013/964149

**Published:** 2013-04-28

**Authors:** Andrzej K. Kuropatnicki, Ewelina Szliszka, Wojciech Krol

**Affiliations:** ^1^Pedagogical University of Krakow, Karmelicka 41, 31-128 Krakow, Poland; ^2^Department of Microbiology and Immunology, Medical University of Silesia in Katowice, Jordana 19, 41-808 Zabrze-Rokitnica, Poland

## Abstract

Propolis (bee glue) has been known for centuries. The ancient Greeks, Romans, and Egyptians were aware of the healing properties of propolis and made extensive use of it as a medicine. In the middle ages propolis was not a very popular topic and its use in mainstream medicine disappeared. However, the knowledge of medicinal properties of propolis survived in traditional folk medicine. The interest in propolis returned in Europe together with the renaissance theory of *ad fontes*. It has only been in the last century that scientists have been able to prove that propolis is as active and important as our forefathers thought. Research on chemical composition of propolis started at the beginning of the twentieth century and was continued after WW II. Advances in chromatographic analytical methods enabled separation and extraction of several components from propolis. At least 180 different compounds have been identified so far. Its antibacterial, antiseptic, anti-inflammatory, antifungal, anesthetic, and healing properties have been confirmed. Propolis has been effectively used in treatment of dermatological, laryngological, and gynecological problems, neurodegenerative diseases, in wound healing, and in treatment of burns and ulcers. However, it requires further research that may lead to new discoveries of its composition and possible applications.

## 1. Introduction

Propolis, or bee glue, is a natural wax-like resinous substance found in bee hives where it is used by honeybees as cement and to seal cracks or open spaces. At elevated temperatures propolis is soft, pliable, and very sticky; however, when cooled, and particularly when frozen or at near freezing, it becomes hard and brittle. It will remain brittle after such treatment even at higher temperatures. Typically propolis will become liquid at 60 to 70°C, but for some samples the melting point may be as high as 100°C [1]. Early observers of bee behaviour were aware of plant origin of propolis, the fact that was asserted by Philipp [2] and Vansell and Bisson [3]. It is now generally accepted that propolis is collected by honeybees from tree buds or other botanical sources in the North Temperate Zone, which extends from the Tropic of Cancer to the Arctic Circle. The best sources of propolis are species of poplar, willow, birch, elm, alder, beech, conifer, and horse-chestnut trees [4]. Its colour varies from green to brown and reddish, depending on its botanical source. Honeybees have been observed collecting the protective resins of flower and leaf buds with their mandibles and then carrying them to the hive on their hind legs. Many authors have described the collection and delivery of propolis [4–8]. A colony of bees collects from 150 to 200 g of propolis in one year; however, some races collect less than that [4]. Foraging for propolis is only known with the Western honeybee or European honeybee (*Apis mellifera*) [1], which is a species of bee universally managed by beekeepers. This species has several subspecies or regional varieties, such as the Italian bee (*Apis mellifera ligustica*), European dark bee (*Apis mellifera mellifera*), and the Carniolan honey bee (*Apis mellifera carnica*) [9]. Interestingly, tropical honeybees (*Apis cerana*, *Apis florae,* and *Apis dorsata*) and African *Apis mellifera* make no use of propolis [10].

## 2. The History of Honeybees

The history of bees and their products can be traced back to c. 13,000 BC. A certain amount of knowledge is attested by depictions of the bee and of hive beekeeping found during excavations. Rock paintings also provide some of the earliest evidence of gathering honey from wild colonies. At some point humans began to domesticate wild bees in hives made from hollow logs, wooden containers, pottery vessels, and woven straw baskets. Although no written descriptions of bees and beekeeping are known from ancient Egypt, archaeological excavations attest that honeybees were kept there and that the main centre of beekeeping was lower Egypt with its extensive irrigated lands full of flowering plants. From c. 3100 BC the honeybee was used as a hieroglyph in the topographical symbol of Ancient Egypt [11]. Since earliest times the gods were associated with the bee and one of the pharaohs' titles was “Bee King” [12]. Temples kept bees in order to satisfy the desire of the gods for honey and for the production of medicines and ointments. According to da Silva Veiga, honey was known to the priests and it was used in the embalming process as well as for conservation purposes [13]. The evidence for such usage is; however, scant and anecdotal. For example, Ernest Budge in his book *The Mummy* presents an unsupported tale of Abd el-Latif about treasure hunters who found a sealed jar containing honey, nd after eating part of it they discovered that it also contained the body of a small child [14].

A system of high-status apiculture existed in ancient Greece. The most ancient personality, related to apiculture, presented in historical documents, is Aristaios, son of god Apollo and nymph Cyrene. Regarded as the father of apiculture he is one of the most enigmatic figures of ancient Greek religion. The muses taught him among other skills the art of beekeeping and then Aristaios passed his knowledge on to the mortals. In Knossos archaeologists discovered hives, smoking pots, honey extractors, and other beekeeping paraphernalia [15]. Also a number of written records survived. Aristotle's *Historia animalium *(*History of Animals*) is divided into ten books. The book IV discusses animals without blood and in the book V, Chapter XVIII, Aristotle makes a number of remarkable observations about bees [16]. Roman writer Virgil writes about “Heaven's gift, the honey from the skies” [17], and many other Latin writers like Pliny the Elder [18], Rutilius Palladius [19], and Marcus Varro [20] left the descriptions of bees and beekeeping in ancient Rome. Lucius Junius Moderatus Columella authored *De Re Rustica*, the book IX of which is devoted to “Wild animals—Bees, the management of them, their diseases and pests, honey and wax” [21]. Gaius Julius Hyginus, who flourished in the 1st century AD, wrote on agriculture and beekeeping. However, of his numerous works nothing has survived.

In the Quran there is a long chapter (*sorat*) with the name of bees which says about honey being healing for man:


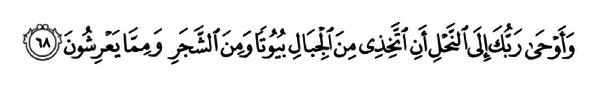

*And your Lord inspired to the bee, Take for yourself among the mountains, houses, and among the trees and [in] that which they construct.*

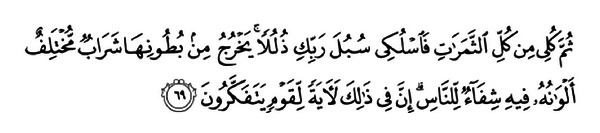

*Then eat from all the fruits and follow the ways of your Lord laid down [for you]. There emerges from their bellies a drink, varying in colors, in which there is healing for people. Indeed in that is a sign for a people who give thought *[22]. **

## 3. Propolis

The word “propolis” derives from Hellenistic Ancient Greek $\pi \rho \overset{´}{o}\pi o\lambda ı\varsigma$ (suburb, bee glue) which originates from Greek verb $\pi \rho o\text{-}\mu \overset{˘}{\alpha }\lambda \overset{´}{\alpha }\sigma \sigma \omega$ (promalasso) Att. $\pi \rho o\mu \overset{˘}{\alpha }\lambda \overset{´}{\alpha }\text{-}\tau \tau \omega$, “soften beforehand, make supple by rubbing or kneading” (Aristotle, *Problemata* 869b30) [23]. It is defined by Lewis and Short as “the third foundation in making honey, a gummy substance with which the bees close the crevices of their hives, bee-glue” [24]. It has been generally believed that honeybees produce propolis to help protect the hive. Apart from its role in sealing holes, blocking cracks, and smoothing out the internal walls, bee glue appears to act as an antiseptic to prevent microbial infection of larvae, honey stores, and the combs. As Seeley and Morse noted, bees apply propolis to areas where combs are to be attached thus creating smooth and germ-free surfaces. Because honeybee populations are so confined and live in close contact, illness in one bee can spread quickly to the whole hive. Yet hives stay healthy because the bees manufacture their own antibiotic, that is, propolis, which reduces microbial growth on hive walls. What is more, propolis protects the hive against uncontrolled airflow and external moisture. The thin layer of propolis provides an impermeable lining which limits the escape of water and maintains constant humidity inside the hive [25, 26]. 

## 4. Early History of Propolis

Propolis is as old as honey, and it has been used by man for ages. There are records suggesting the use of it by ancient Egyptians, Persians, and Romans [27]. Ancient Egyptians depicted propolis-making bees on vases and other ornaments and used it to alleviate many ailments [28]. The Egyptians had learnt from the bees, which use propolis as an “embalming” substance. The bees cover the carcass of an invader, which was killed but could not be transported out of the hive, with propolis and wax [29]. In this way the bees restrain spread of infection caused by decomposing carcass. In the 1960s, Derevici et al. showed that propolis is responsible for the lower incidence of bacteria within the hive [30]. 

The ancient Jews considered *tzori* (the Hebrew word for propolis) as a medicine. *Tzori* and its therapeutic properties are mentioned throughout the Old Testament [31]. The biblical Balm of Gilead (*tzori Gilead* in Hebrew) is nearly indistinguishable from propolis. Balm of Gilead is described in the Bible as the gift that the Queen of Sheba gave to King Solomon. In Judea, it was grown around the dead sea for about 1,500 years and achieved fame due to its aroma and medicinal properties. It is made of resin from various poplars, including *P. balsamifera*, *P. nigra*, and *P. gileadensis* [32]. Balm of Gilead was one of the several components of the special incense that was used twice daily in the Holy Temple in Jerusalem. The identification of the balm of Gilead with the Hebrew names Afarsemon, kataf, nataf, and tzori Gilead can be traced to several sages, including Shimon Ben-Gamliel, Rambam (Maimonides), Saadia Gaon, and the modern biblical botanist Yehuda Feliks [33]. 

The Greeks used propolis as the primary ingredient of *polyanthus*, perfume which combined propolis, olibanum, styrax, and aromatic herbs [34]. More than 15 Greek and Roman authors reported on the preparation and application of propolis, the so-called third natural product of bees (beside honey and wax). In *Historia Animalium *in book IX (Books I–VIII were written by Aristotle; the author of Book IX is anonymous) one can find the following characteristics of propolis:

 When the hive has been delivered to them [honey bees] clean and empty, they build their waxen cells, bringing in the juice of all kinds of flowers and the “tears” or exuding sap of trees, such as willows and elms and such others as are particularly given to the exudation of gum. With this material they besmear the groundwork, to provide against attacks of other creatures; the bee-keepers call this stuff “stop-wax”. They also with the same material narrow by side-building the entrances to the hive if they are too wide [35].

And then the author continues

 At the entry to the hive the aperture of the doorway is smeared with mitys; this substance is a deep black, and is a sort of dross or residual by-product of wax; it has a pungent odour, and is a cure for bruises and suppurating sores [36].

Hippocrates is said to have used propolis to cure wounds and ulcers, both external and internal [37]. Pedanios Dioscorides, who lived around 50 AD, described medical uses of propolis in his chief work *De materia Medica*. Apart from propolis, he often mentions honey, wax, and various honey wines as a medicine [38]. Dioscorides wrote about propolis:

 the yellow bee glue that is of a sweet scent and resembling styrax is to be chosen and which is soft and easy to spread after the fashion of mastic. It is extremely warm and attractive and is good for the drawing out of thorns and splinters. And being suffimigated it doth help old coughs and being applied it doth take away the lichens [39].

The Romans also revered the bee and propolis extensively. Pliny the Elder in his famous *Natural History* writes that

 The propolis is produced from the sweet gum of the vine or the poplar, and is of a denser consistency, the juices of flowers being added to it. Still, however, it cannot be properly termed wax, but rather the foundation of the honey-combs; by means of it all inlets are stopped up, which might, otherwise, serve for the admission of cold or other injurious influences; it has also a strong odour, so much so, indeed, that many people use it instead of galbanum [40].

Pliny also describes the practical usage of this substance. According to him “[propolis] has the property of extracting stings and all foreign bodies from the flesh, dispersing tumours, ripening indurations, allaying pains of the sinews, and cicatrizing ulcers of the most obstinate nature.” [41]. 

According to Marcus Terentius Varro,

 Propolis is the name given to a substance with which they build a *protectum* (“gable”) over the entrance opening in front of the hive, especially in summer. This substance is used, and under the same name, by physicians in making poultices, and for this reason it brings even a higher price than honey on the Via Sacra [20].

In the first century AD, Cornelius Celsus wrote about propolis as a drug for promoting suppuration, for opening wounds, and for treatment of abscesses. In *De Medicina *Celsus writes: “The following mature abscessions and promote suppuration: nard, myrrh, costmary, balsam, galbanum, propolis, storax, frankincense, both the soot and the bark, bitumen, pitch, sulphur, resin, suet, fat, oil.” [42]. 

Arabs may have known propolis as well. For instance, Avicenna wrote about two different kinds of wax, that is, clean wax and black wax, the latter being probably propolis. He says “by its strong smell it makes you sneeze” and “[it] has the characteristics to eliminating the spikes of the bolts and the stakes. It also rarefies, cleans and soaks” [39]. In the Persian manuscripts propolis is described as a drug against eczemas, myalgia, and rheumatism. 

## 5. Propolis in Medieval Times

In the Middle Ages propolis was not a very popular topic and its use in mainstream medicine soon disappeared. Only few manuscripts dealing with propolis have survived. Some sources from the twelfth century describe medicinal preparations containing bee glue which were used for treatment of oral and pharyngeal infections as well as dental caries. In the Georgian original medical treatise dated to c. 1486 *Karabadini* (*Book of Medical Treatment*), the author suggests that propolis is good against dental decay [39]. *Karabadini* was authored by Zaza Panaskerteli-Tsitsishvili, a Georgian prince, politician, and man of letters. His treatise builds upon anonymous Georgian compendia of Galenic medicine, notably the 11th century *Ustsoro Karabadini* (*Peerless Handbook*) and the 13th century *Tsigni Saakimoy* (*Doctoring Book*). The work summarizes the state of medical knowledge in Georgia and neighboring cultures at that time [43]. Although earlier books describing medical application of propolis claim the efficacy of this bee product, they do not contain any detailed instructions on the preparations nor any sources of information [44, 45].

Fortunately, the knowledge of medicinal properties of propolis survived in traditional folk medicine and, what is more, propolis was still extensively used in “herbal” medicine on the territories of Eastern Europe. Significantly, propolis has been often called “Russian penicillin” [46]. Arab merchants mentioned abundance of bees and honey on Slav soil in the 9th and 10th centuries, and the first reference to Polish keepers of wild forest bees comes from the beginning of the 11th century. A diplomat and merchant from the Moorish town of Tortosa in Al-Andalus, known under his Arabic name Ibrahim ibn Jakub in the year 965, wrote “The land of Meshko [father of Boleslav Khrobry, the first crowned king of Poland] is rich in grain and meat and honey and fields” [47]. According to Herbord, bishop of Levant, in Pomerania “there is a great abundance of honey and wheat, of hemp and of poppies and of all kinds of vegetables” [48]. 

## 6. Propolis in Early Modern Times

The interest in propolis returned in Europe together with the Renaissance theory of *ad fontes*, which brought back an interest in ancient teaching and medicine. Thanks to medical humanists some old and forgotten remedies and treatments were rediscovered and used over again. John Gerard in his famous herbal book, *The History of Plants* (1597), makes reference to the use of “the resin or clammy substance of the black poplar tree buds” for healing ointments. “The ointment that is made of the [poplar] buds, is good against all inflammations, bruses, squats, fals, and such like” [49]. Propolis is found to be included in pharmacopoeias in England in the seventeenth century as a major ingredient of healing ointments [50]. Nicholas Culpeper, botanist and physician, in his *Complete Herbal* under the heading “The poplar tree” states that “the ointment called Populneon, which is made of this Poplar, is singularly good for all heat and inflammations in any part of the body, and tempers the heat of wounds. It is much used to dry up the milk of women's breasts when they have weaned their children” [51]. In *The Universal Herbal* published in 1824, one can read under “Populus Nigra; Black Poplar Tree”:

 The young leaves are an excellent ingredient for poultices for hard and painful swellings. The buds of both this and the White Poplar smell very pleasantly in the spring, and, being pressed between the fingers, yield a balsamic resinous substance [propolis], which extracted by spirits of wine, smells like storax. A drachm of this tincture in broth is administered in internal ulcers and excoriations and is said to have removed obstinate fluxes proceeding from an excoriation of the intestines [52].

## 7. Early Research on Propolis

At the beginning of the 19th century propolis was studied and described by Nicolas Louis Vauquelin, a French pharmacist and chemist. In the report made to the Society of Agriculture Vauquelin writes that propolis or bee mastic is collected by the bees. It is resinous, ductile, odorant substance of a reddish brown colour. “In the mass it is blackish; but it is semitransparent when in thin plates. The heat of the fingers is sufficient to soften it and give it all the ductility of wax, but it is more tenacious.” Like wax it may be chewed between the teeth and is tasteless. “Its odor is aromatic, resembling that of *meliloti herba*, of balsam of Peru, or of the Banana poplar.” Vauquelin used 100 g of propolis which was digested three times in alcohol and filtered each time. The last addition of alcohol was followed by boiling the substance for a few minutes. Finally, to get rid of the fat matters it retained from fragments of bees, as well as some vegetable substances and grains of sand, boiling diethyl ether was poured on it, and the mass was pressed through a fine strainer. The residue dried, weighed 14 g. The component parts calculated afterwards present as follows: pure wax 14 g, pure resin of propolis 57 g, extraneous bodies 14 g, loss, acid, aroma 15 g. This resinous mass was pure propolis, which melts readily on the fire; it yields by distillation a volatile oil, which is white and of a very agreeable smell. The fixed part acquires a deeper color and becomes harder; it is soluble in fixed and volatile oils [53].

The development of research on propolis was strictly connected with the development of chemistry. Examples include studies of the chemistry of flavonoids, common compounds contained in propolis. Flavonoids are a diverse group of phytochemicals that are produced by various plants in high quantities. Based on their skeleton, flavonoids are classified into eight groups: flavans, flavanones, isoflavanones, flavones, isoflavones, anthocyanidines, chalcones, and flavonolignans. Flavones, a class of flavonoids, are the most important plant pigments for flower coloration producing yellow or red/blue pigmentation. Early in the nineteenth century, in 1814 or 1815, Michel Eugène Chevreul, a French chemist, obtained several flavones in the crystalline state: morin from *Maclura tinctoria* (old fustic), luteolin from *Reseda luteola *(weld), fisetin from *Rhus cotinus* (young fustic), and quercitrin from *Quercus tinctoria* (quercitron bark). Chrysin (C_15_H_10_O_4_), a very weak coloring matter, was the first flavone to be isolated in the pure state from the buds of the common poplar by Piccard in 1864 [54]. In 1879 Carl Liebermann prepared quercetin from quercitrin but assigned to it an erroneous formula. The structures of fisetin, the coloring matter of young fustic, and of quercetin were elucidated by Austrian chemist Herzig in 1891 [55]. 

In 1893 Stanislaw Kostanecki, Polish professor working at the University of Bern, Switzerland, found out that phenyl derivative of benzopyrene is a natural substance of chrysin [56]. In 1895 Kostanecki submitted chrysin to examination. Shortly thereafter Kostanecki proved the constitution of chrysin, and he also gave the names flavone (from Latin *flavus*, yellow) and flavonol to the parent ring system and its 3-hydroxy derivative. From the period of 1895 onwards, a considerable number of natural yellow coloring matters were examined, many of which have been proved to belong to the flavone or flavonol groups, and there can be no doubt that of all the natural dyes, these are much the most widely distributed in nature [57–60]. Between 1895 and 1920 Perkin investigated numerous plant materials containing flavones. In 1924 Robinson described a general reaction, which has been widely used for synthesis in this field. More recently, a major contribution to the knowledge of natural flavones has been made by Seshadri [61] and Seshadri and Venkateswarlu [62].

In 1898 Emilewicz, Kostanecki, and Tambor announced the synthesis of chrysin, employing for this purpose a series of reactions which represent a reversal of the scheme of hydrolysis. Other methods of synthesis were subsequently applied to chrysin by Kostanecki and his coworkers, and in 1899 flavone itself was prepared, followed in 1900 by apigenin (parsley) and luteolin (weld). Somewhat later, a method was devised for the artificial preparation of flavonols, and in 1904 fisetin, quercetin and kaempferol were synthesized by Kostanecki and his coworkers, morin being similarly obtained in 1907. The flavonols, with the exception of morin, which is colorless, are yellow crystalline substances, soluble in alkaline solutions with yellow color, and yield with ease in the presence of acetic acid orange crystalline oxonium salts [63]. Kostanecki also determined the structure of curcumin and studied brazilein, hematoxylin, and cochineal [64]. Having analyzed nearly 2000 various substances Kostanecki showed that 200 of them contain flavone derivatives. 

## 8. Studies on Composition of Propolis

Research on chemical composition of propolis started at the beginning of the 20th century. Early attempts to determine the composition of propolis were concerned with simple fractionation. One of the earliest reports is that of Dieterich and Helfenberg in which they present their extraction methods and propolis constituents separated in alcohol, chloroform, and ether [65, 66]. In 1911, in his later work, Dietrich identified vanillin in propolis [67], and another German researcher working on propolis Küstenmacher identified cinnamic acid and cinnamyl alcohol as components of propolis [68]. In 1926, Jaubert identified pigment chrysin, a naturally occurring flavone in bee glue and also showed that chrysin gives color to beeswax [69]. In 1927 a German scientist Rösch confirmed the hypothesis of Plinius that propolis originates from the buds of plants [70]. A series of studies conducted in the USA led to the detection of small amounts of vitamins B1, B2, B6, C, and E as well as nicotinic acid and pantothenic acid in propolis [71–73]. In 1957, Ushkalova found four types of wax in propolis, all varying in color [74]. Before 1967 a series of studies were conducted on the physiological action and therapeutic uses of flavonoids [75]. Powers found that all flavonoids studied by him showed inhibitory activity towards one or more of ten bacteria strains [76].

The studies on chemical composition of propolis were continued in the 1960s. At first, propolis was thought to be of very complex, but rather constant chemistry, like beeswax [77]. Later, however, the analysis of numerous samples from different geographic regions as well as application of advanced laboratory methods showed that the chemical composition of bee glue is highly variable. In 1969 Popravko together with others separated and identified two flavanones and isovanillin as well as six flavonoid pigments in propolis [78, 79]. After Lavie had demonstrated that propolis shows antibacterial activity towards *Bacillus subtilis*, *Bacillus alvei,* and *Proteus vulgaris* [80] French scientists managed to isolate from propolis extracts the flavon galangin, which was found to be partly responsible for this activity [81]. Later the same team isolated and identified pinocembrin, tectochrysin and isalpinin [82]. In 1970 Cizmarik and Matel reported separation and identification of 3,4-dihydroxycinnamic acid and 4-hydroxy-3-methoxycinnamic acid which are present in propolis [83, 84]. Nikiforov with coworkers detected copper and manganese in propolis [85], and at the same time Herold examined ash residue of propolis and found iron, calcium, aluminum, vanadium, strontium, manganese, and silicon in it [86]. 

In the 1970s, advances in chromatographic analytical methods, such as column chromatography and thin layer chromatography, allowed for separation and extraction of more components from propolis. In 1975 Schneidweind and coworkers identified 17 constituents of propolis, including 9 previously identified compounds [87]. Simultaneously, Metzner with coworkers using bioautographic methods proved that only a few compounds detectable in the extracts of propolis have significant antimycotic activity [88]. In 1977, Australian researchers separated and identified four flavones, pinostrobin, sakuranetin, isosakuranetin, pterostilbene, chrysin, 3,5-dimethoxybenzyl alcohol, and xanthorrhoeol [89]. In 1979  Vanhaelen and Vanhaelen-Fastre used gas chromatography (GC) and high-performance liquid chromatography (HPLC) in propolis analysis. Application of gas chromatography mass spectrometry (GC-MS) led to identifying sugars in propolis. Marcucci and Bankova et al. have registered over 300 known substances in propolis [90, 91]. Heinen and Linskens studied fatty acid constituents of propolis. They showed that fatty acid fractions contain C_7_–C_18_ acids [92]. Popravko presented 18 chemical components of propolis, 14 of which belong to flavonoid compounds [93]. 

The composition of propolis is not fixed and varies considerably from region to region along with vegetation, from season to season, and from hive to hive. In each sample of propolis, more than 80 to 100 chemical compounds are typically identified [94]. Altogether, at least 180 different compounds have been identified in propolis so far. A broad analysis revealed approximately 50 constituents in “typical” European propolis, which comes usually from trees, such as poplars and conifers. These constituents comprise primarily resins and vegetable balsams, mainly cinnamic acid and derivatives, coumaric acid, prenylated compounds, artepillin C (50%), beeswax (30%), essential oils (10%), bee pollen (5%), and minerals, polysaccharides, proteins, amino acids, amines, amides, and organic debris (5%) [95]. The major constituents of propolis from most of the sources are flavonoids [4]. Some of the principal phenolic esters and flavonoids like caffeic acid phenethyl ester, quercetin, baicalin, pinocembrin, naringin, galangin, and chrysin have been found to be responsible for antimicrobial, antioxidant, and anti-inflammatory activities of propolis [96].

In tropical regions bees also gather resin from flowers in the genera *Clusia *and *Dalechampia*, in addition to a large variety of trees. These are the only known plant genera that produce floral resins to attract pollinators [97–101]. As Ghisalberti noted in his review of propolis, many compounds isolated so far from propolis represent a fraction of its content. Most of them come from the part of propolis soluble in organic solvents, whereas the large part of bee glue which is not readily soluble in water or organic solvents most probably consists of natural polymeric material [4].

## 9. “Dr Propolis”

In the years 1967–1973 a series of studies were performed in Denmark, the results of which turned to be sensational. The effectiveness of propolis in treatment was proved as well as the fact that it produces almost no sideeffects. Dr Karl Lund Aagaard, a Danish biologist, earned the name “Dr Propolis” for his exploits of over 20 years of propolis collecting and research. After observing the effects of propolis on more than 50,000 patients in Scandinavia, Dr Aagaard drew the following conclusions:

 The field of influence of Propolis is extremely broad. It includes cancer, infection of the urinary tract, swelling of the throat, gout, open wounds, sinus congestion, colds, influenza, bronchitis, gastritis, diseases of the ears, periodontal disease, intestinal infections, ulcers, eczema eruptions, pneumonia, arthritis, lung disease, stomach virus, headaches, Parkinson's disease, bile infections, sclerosis, circulation deficiencies, warts, conjunctivitis and hoarseness [102].

In 1976 Aagaard patented a method for purifying and separating propolis derived from beehives. The method included the steps of quick-freezing untreated propolis repeatedly at temperatures below −20°C and then crushing the treated propolis to smaller particles at a temperature below 10°C. Next, the said particles were separated to a number of fractions according to size, and the fractions containing most impurities were dissolved and filtered in a fluid filter for full utilization of all propolis present. Aagaard discovered that it is not necessary to extract the individual antibiotics from the propolis but that the substance in its natural form has a powerful curative effect on various diseases such as chronic colitis, pharyngitis, rheumatism, and conjunctivitis [103].

## 10. Antibacterial Properties of Propolis

The earliest systematic investigation of the antibacterial activity of propolis was performed by Kivalkina in the 1940s. Propolis was shown to have bacteriostatic activity against *Streptococcus aureus*, the typhoid bacillus, and some other bacteria as well [104]. The antimicrobial activity of propolis was also studied by Lindenfelser. In 1967 he examined propolis samples collected in various parts of the USA at different seasons. Of 39 bacterial species that were tested *in vitro* 25 proved to have strong inhibitory activity, whereas of the same number of fungus species 20 were inhibited [77]. Antibacterial properties of propolis against a wide spectrum of bacteria were studied by Scheller in Poland. In 1967 a series of experiments was performed in which propolis was extracted with ethanol, and after evaporation of alcohol the fraction obtained was dispersed in Tween 20 saline solution. Two other fractions were obtained by extracting propolis with saline solution at 37°C and 100°C. It was shown that the *Coccaceae* were sensitive, whereas *Candida* and *Corynebacterium* strains were partly sensitive to propolis fractions. In the group of mycobacteria, H_37_Rv and the strain isolated from the patient were found to be sensitive to the fraction obtained by alcohol extraction [105]. Several studies in which crude solutions of propolis were tested against a wide range of bacteria were carried out by Germans [106]. 

## 11. Anesthetic Properties of Propolis

In the 1950s it was proved in the experiments on animals that the propolis extract acted as a surface anesthetic with slight penetrating power and that it can be used in dental practice [107, 108]. Todorov and others proved that propolis has an infiltrate action equal to that of procaine [109]. Later on a Bulgarian researcher Tsacov showed that 5% procaine solution of propolis presented a better and quicker action than aqueous alcoholic extract of propolis [110]. Paintz and Metzner in experiments with an ethanol propolis extract and some constituents isolated from propolis tested on the cornea of the rabbit and of the mouse obtained total anesthesia with the total extract as well as with the compounds 5,7-dihydroxyflavanone (pinocembrin), 5-hydroxy-7-methoxyflavanone (pinostrobin) and with a mixture of caffeic acid esters. Each of these compounds was nearly thrice as potent as the total extract [111].

## 12. Application of Propolis

Propolis is reputed to have antiseptic, antibacterial, antimycotic, astringent, spasmolytic, anti-inflammatory, anaesthetic, antioxidant, antitumoural, antifungal, antiulcer, anticancer, and immunomodulatory effects. It has been used in a variety of applications, which include ointments and creams used in wound healing, treatment of burns, skin problems, and ulcers. Various propolis preparations have been applied in treatment of laryngological problems, gynecological diseases, asthma, and diabetes. Propolis has been used in toothpaste and mouthwash preparations to treat gingivitis and stomatitis [112–114]. Antiviral properties of propolis have been known for many years. In studies on *Herpes simplex virus* infection, *in vitro*: 0.5% propolis extract caused 50% inhibition of HSV infection, whereas *in vivo*: as little as 5% propolis prevented the appearance and development of symptoms of HSV-1 infection in animals [115]. Also studies on propolis application in genital herpes infection (HSV type 2) prove its effectiveness [116].

It has been known for a long time that propolis and its extracts have a positive effect on tissue regeneration. Slovak researchers proved that application of an alcoholic solution of propolis as well as framykoin accelerates the tissue regeneration process [117]. It was Scheller from Poland who popularized the term EEP: ethanol extract of propolis. In the 1970s a series of experiments was performed with use of EEP, in which 19 elements were found. Scheller showed that EEP solutions maintained their antibacterial activity in acidic or neutral pH [118]. Later Polish researchers from Scheller's team showed that application of ethanol extract of propolis (EEP) promotes the healing processes in damaged cartilage [119] as well as enhances ossification in artificially induced bone defects [120]. It was demonstrated that EEP supports regeneration of dental pulp and reduces inflammatory and degenerative processes as well [121]. 

Propolis and its extracts have found numerous applications in treatment of various diseases. Aripov et al. used propolis in treatment of experimental stomach ulcers in rats [122]. In the 1970s Gorbatenko applied a 30% alcohol solution of propolis to treat ulcers in patients [123] and Makarov described propolis treatment of ulcers and pyloroduodenitis [124]. Another study was conducted in the Soviet Union by Lutsenko and Pisarenko who applied water-soluble propolis in the treatment of trophic ulcers of the lower extremities in arteriosclerosis obliterans [125]. Damyanliev and others presented positive results of the treatment of suppurative surgical wounds with propolis [126]. In 1986 Korochkin and Poslavskiĭ used propolis in the treatment of chronic gastroduodenal ulcers [127]. 

In orthopedics propolis preparations were applied to bones in the cases of purulent inflammation thanks to which inflammatory process was inhibited and osseous tissue was restored [128]. Propolis was proven to be useful in dental pulp regeneration [121]. Propolis extracts have been widely used in dentistry: in postextraction complications (dry socket), in the treatment of mucous membranes and gingivae. There were also attempts in applying propolis into the treatment of caries and its complications [129], in the treatment of dentinal hypersensitivity [130], in deep parodontopathies, and in the treatment of oral candidiasis [131]. Propolis has been marketed as a treatment for rheumatism and sprains. In the cases of rheumatic diseases articular injections of saline EEP solutions were used. In result pain, edema, and fever disappeared and during long-term treatment pathological process retreated [132]. Propolis also proved to be effective in the treatment of common cold [133] and chronic tonsillitis [134].

The flavonoids and antioxidant phenols concentrated in propolis are powerful antioxidants and have been shown to be capable of scavenging free radicals which can extensively interfere with normal cell metabolism. They thereby protect lipids and other compounds such as vitamin C from being oxidized or destroyed. Active free radicals, together with other factors, are considered to be responsible for cellular ageing and degradation in such conditions as cardiovascular diseases (leading to heart attacks and strokes), arthritis, cancer, diabetes, and neurodegenerative diseases such as Alzheimer disease [135–139]. Propolis has also been used in cosmetic products, such as face creams, ointment, lotions, and solutions. The properties of propolis have been widely discussed in numerous review papers [140–147].

## 13. Conclusion

Propolis is a natural product that has been known and used by man for centuries. It is mainly because man learnt relatively early to exploit the products of domesticated honeybee. Recorded use of propolis dates back to c. 300 BC and continues today in the form of home remedies, toothpastes, creams, ointments, drops, and dietary supplement. Its numerous properties have been appreciated for very long time. However, despite numerous studies conducted all over the world so far, the constitution of propolis remains largely unknown. It requires further research that may lead to new discoveries of its composition and possible applications. 
